# Overall Assay of Neuronal Signal Propagation Pattern With Long-Term Potentiation (LTP) in Hippocampal Slices From the CA1 Area With Fast Voltage-Sensitive Dye Imaging

**DOI:** 10.3389/fncel.2018.00389

**Published:** 2018-10-24

**Authors:** Yoko Tominaga, Makiko Taketoshi, Takashi Tominaga

**Affiliations:** Laboratory for Neural Circuit Systems, Institute of Neuroscience, Tokushima Bunri University, Sanuki, Japan

**Keywords:** voltage-sensitive dye, hippocampus, LTP, optical recording, L-LTP, HFS, TBS

## Abstract

Activity-dependent changes in the input-output (I-O) relationship of a neural circuit are central in the learning and memory function of the brain. To understand circuit-wide adjustments, optical imaging techniques to probe the membrane potential at every component of neurons, such as dendrites, axons and somas, in the circuit are essential. We have been developing fast voltage-sensitive dye (VSD) imaging methods for quantitative measurements, especially for single-photon wide-field optical imaging. The long-term continuous measurements needed to evaluate circuit-wide modifications require stable and quantitative long-term recordings. Here, we show that VSD imaging (VSDI) can be used to record changes in circuit activity in association with theta-burst stimulation (TBS)-induced long-term potentiation (LTP) of synaptic strength in the CA1 area. Our optics, together with the fast imaging system, enabled us to measure neuronal signals from the entire CA1 area at a maximum frame speed of 0.1 ms/frame every 60 s for over 12 h. We also introduced a method to evaluate circuit activity changes by mapping the variation in recordings from the CA1 area to coordinates defined by the morphology of CA1 pyramidal cells. The results clearly showed two types of spatial heterogeneity in LTP induction. The first heterogeneity is that LTP increased with distance from the stimulation site. The second heterogeneity is that LTP is higher in the stratum pyramidale (SP)-oriens region than in the stratum radiatum (SR). We also showed that the pattern of the heterogeneity changed according to the induction protocol, such as induction by TBS or high-frequency stimulation (HFS). We further demonstrated that part of the heterogeneity depends on the I-O response of the circuit elements. The results show the usefulness of VSDI in probing the function of hippocampal circuits.

## Introduction

Brain function depends on neural circuit activity. Understanding the circuit behavior of *in vitro* specimens is essential to address the physiology and pathology of the normal and diseased brain. Conventional electrophysiological methods, such as field potential recordings, sharp electrode intracellular recordings and patch clamping, are useful for determining the activity of the cells in the circuit and the synaptic connections between elements. However, there is increasing demand to directly elucidate the circuit activity, as the excitation/inhibition (E/I) balance of neural circuits has gained attention (Isaacson and Scanziani, 2011). The E/I imbalance should affect the control and synchrony among various circuit elements and cause a diverse range of psychiatric disorders, including autism spectrum disorders (ASDs; Persico and Bourgeron, 2006), schizophrenia (Canitano and Pallagrosi, 2017) and Alzheimer’s disease (AD; Busche and Konnerth, 2016). One must evaluate the activity across the broad span of the circuit to understand neural circuit mechanisms of brain malfunction (Uhlhaas and Singer, 2012; Anticevic and Murray, 2017).

Optical recordings of membrane potential changes in neurons could be an ideal measurement technique to achieve this goal. Optical recording began with a single point observation of the optical characteristic changes by membrane excitation (Hill and Keynes, 1949; Cohen et al., 1968, 1970), which later led to an imaging technique that employed synthetic voltage-sensitive dye (VSD; Davila et al., 1973; Ross et al., 1974) that could capture the real-time activity of brain circuit function in the 1980’s (Grinvald et al., 1981, 1982, 1988; Ichikawa et al., 1993; Vranesic et al., 1994). Wide-field large-scale voltage imaging can probe the circuit mechanism in animal models of healthy and disease states (Tanemura et al., 2002; Mann et al., 2005; Suh et al., 2011; Juliandi et al., 2016). However, there are several technical challenges that prevent wide-field imaging from meeting experimental requirements, namely, the low sensitivity of the dye and the fast signaling of neurons.

Comparing the voltage dependence of the VSD in a single membrane (~%/100 mV; Loew et al., 1992) to the signal size from the bulk-stained brain tissue, the VSD signal is usually small (10^−2^–10^−3^; Peterka et al., 2011). The low optical signal is due to the ratio of the fluorescence from the membrane with constant (unaffected), and sub-threshold potential change; this is more significant than that produced by a substantial potential-change such as action potential (Tominaga et al., 2009). Additionally, the camera must have a high frame rate that can capture membrane potential events that occur in the millisecond range. The camera should also be able to capture a large amount of light during the limited timeframe to avoid photon-shot noise, i.e., the randomness of the number of photons proportional to the square root of total photons. Ultimately, the camera must fulfil these essential characteristics, i.e., having low noise (at least 60 dB at 10^−3^ change), a high-speed (sub-millisecond) frame rate, and the ability to capture a large amount of light (at least 10^5^ photons). Several commercially available imaging systems can meet these requirements. Optics are also crucial, especially in the low-magnification range, and slice handling is essential to avoid mechanical noise and to maintain proper physiology.

In the present article, we present how our imaging system can be used with hippocampal slices prepared via well-known methodology. We demonstrate an imaging analysis of long-term potentiation (LTP; Bliss and Gardner-Medwin, 1973; Bliss and Collingridge, 1993) in the hippocampal CA1 area, which is a key indicator of brain function in many disease models (Monday and Castillo, 2017; Monday et al., 2018).

## Materials and Methods

### Slice Preparation and Staining With VSD

All animal experiments were performed according to protocols approved by the Animal Care and Use Committee of Tokushima Bunri University. Hippocampal slices (350 μm thick) were prepared from 4- to 5-week-old male mice (C57BL6), decapitated under deep isoflurane anesthesia. The brains were quickly cooled in iced artificial cerebrospinal fluid (ACSF; 124 mM NaCl, 2.5 mM KCl, 2 mM CaCl_2_, 2 mM MgSO_4_, 1.25 mM NaH_2_PO_4_, 26 mM NaHCO_3_ and 10 mM glucose, pH 7.4, after bubbling with mixed 95%/5% O_2_/CO_2_ gas). After cooling for 5 min, the hippocampus was dissected out along with the surrounding cortex and sliced in 350-μm-thick sections with a vibratome (Leica VT-1000 and VT-1200s). Each slice was transferred onto a fine-mesh membrane filter (Omnipore, JHWP01300, 0.45 μm pores, Merk Millipore Ltd., MA, USA) held in place by a thin plexiglass ring (inner diameter, 11 mm; outer diameter, 15 mm, thickness 1–2 mm). Slices placed in the plexiglass ring were transferred to a moist chamber continuously supplied with a humidified mixture of O_2_ and CO_2_ gas. The temperature was held at 28°C for 25 min and then at room temperature. After 1 h of incubation in the chamber, slices were stained with an aliquot of the staining solution (100–110 μl for each slice) of VSD [0.1 mM Di-4-ANEPPS (D-1199, Molecular Probes Inc. OR, USA) in a mixture of 2.7% ethanol, 0.13% Cremophor EL (Sigma), 50% fetal bovine serum (Sigma) and 50% ACSF] for 20 min. The slices were subjected to experiments after at least 1 h of incubation at room temperature.

### Recording

**Figure 1:**
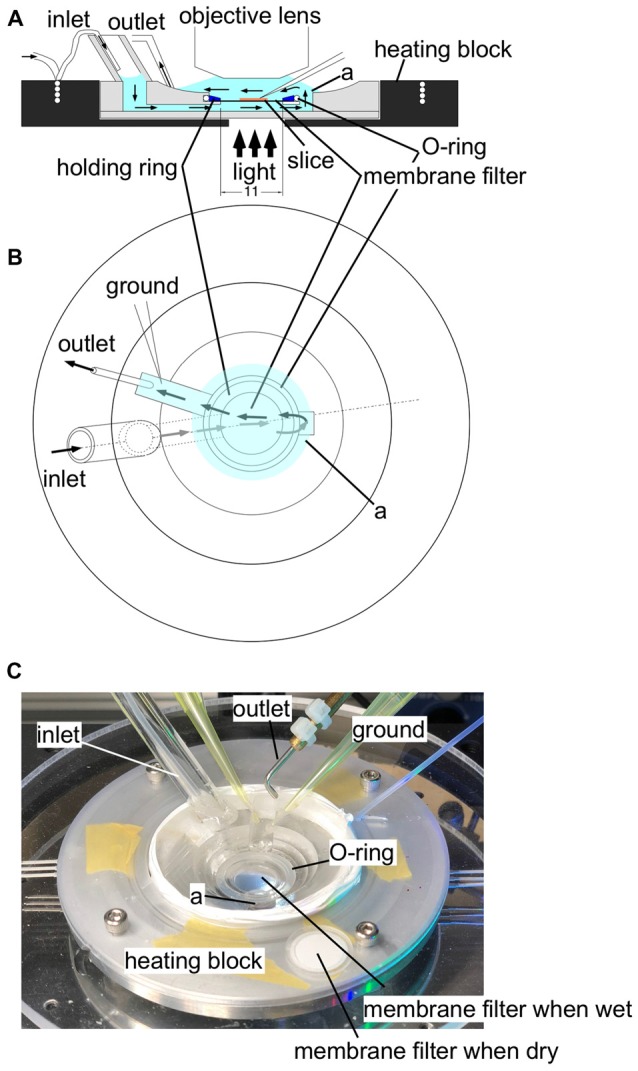
An illustration of the experimental recording chamber system. **(A)** A side view of the recording chamber. An aluminum heating block (black) surrounds the plexiglass recording chamber. A thin tubing embedded in the heating block supplies gassed ACSF to the recording chamber through a wide bubble-trapping tube. The ACSF then flows beneath the specimen held by a polytetrafluoroethylene (PTFE) membrane filter (13 mm in diameter) fixed to a thin (1 mm in height) plexiglass holding ring. The ACSF flows upward through a connecting channel and over the specimen. The tight sealing of the O-ring and the plexiglass holding ring ensures a single stream of ACSF, so that the ACSF supply gas and glucose from both sides of the specimen. The ACSF is then sucked by the outlet tubing. **(B)** A top view of the chamber system. Arrows represent the flow of ACSF. **(C)** A photograph of the experimental recording system. In the center of the recording chamber, a wet membrane filter was placed to show that the membrane filter becomes transparent so that transmitted illumination is possible. A membrane filter was placed outside the recording chamber before use to show the membrane filter when it is dry for comparison.Label (a) shows the well connecting the fluid channel beneath the membrane filter and the top space to cover a slice.

**Figure 2:**
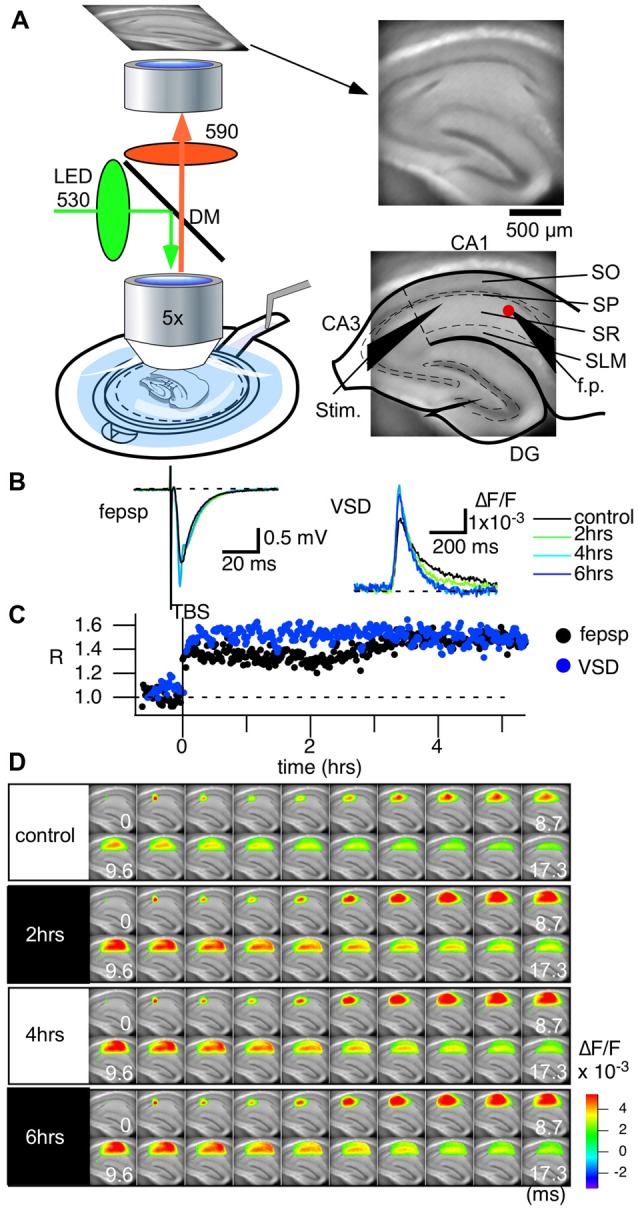
Voltage-sensitive dye (VSD) imaging can follow the long-term circuit activity changes of hippocampal slices caused by theta-burst stimulation (TBS). **(A)** An illustration showing the recording setup of the optical recording system. A membrane filter attached to a thin plastic ring holds a slice preparation. The ring is then held in place with a recording chamber, in which adequate ACSF is provided from the bottom of the experimental chamber and flows over the slice towards the outlet tubing. A water immersion objective lens (5× Olympus, NA 0.35) of a tandem-lens configured epifluorescent optics unit is placed above the slice preparation. An imaging device (MiCAM Ultima and MiCAM02, Brainvision Inc., Tokyo, Japan) captures the fluorescent images of the slice (right). The configuration of the electrodes (Stim., the stimulation electrode; f.p., the field potential electrode) and an illustration of the hippocampus are superimposed. Abbreviations: SO, stratum oriens-alveus; SP, stratum pyramidale; SR, stratum radiatum; SLM, stratum lacunosum-moleculare. **(B)** Traces of field excitatory postsynaptic potentials (fEPSPs) recorded with the field electrode before and after TBS-induced long-term potentiation (LTP) and the optical signal at the corresponding pixel. Both traces are average of eight traces. **(C)** Time course of LTP with the fEPSP slope (black) and the optical signal (blue) at the tip of the field potential electrode. **(D)** The panel shows consecutive images of the response for the control recording and for recordings made at 2 h, 4 h and 6 h. We recorded the optical signals every 60 s at a frame rate of 0.1 ms/frame.

**Figure 3:**
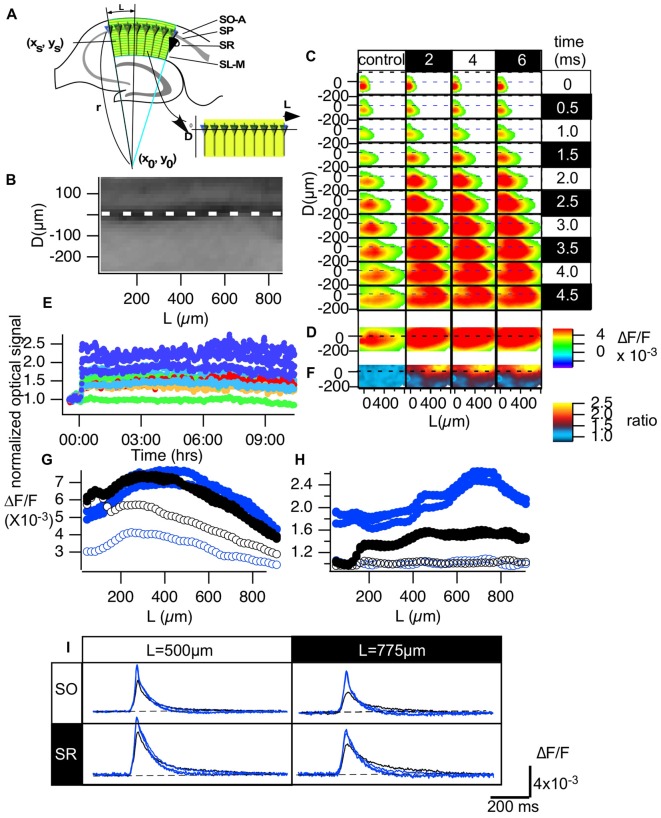
Normalization made it possible to evaluate the optical signals in the CA1 area of hippocampal slices. **(A)** An illustration of how we converted the hippocampal CA1 area to the distance from the start of the CA1 (L) and the distance from the pyramidale cell layer (D) plane **(B)**. **(C)** Representative propagation pattern of the optical signal spread along the CA1 area converted to the D-L plane in each time frame for the control recording and recordings made 2, 4 and 6 h from TBS. **(D)** The peak value of each pixel in the D-L plane. **(E)** The time course of the normalized optical signals in pixels of the D-L plane for 10 h. **(F)** The amplitude of the normalized degree of LTP mapped to the D-L plane at each time point. **(G)** The line profile of the optical signal along the line at *D* = −75 μm (SO) and *D* = 150 μm (SR) at each time point (control, 2, 4 and 6 h). **(H)** The line profile of the normalized degree of LTP along the same lines. **(I)** The time course of the optical signal at the representative points (*L* = 500 and 775 μm, respectively) in each line of the SO (*D* = −75 μm) and the SR (*D* = 150 μm) at each time point.

**Figure 4:**
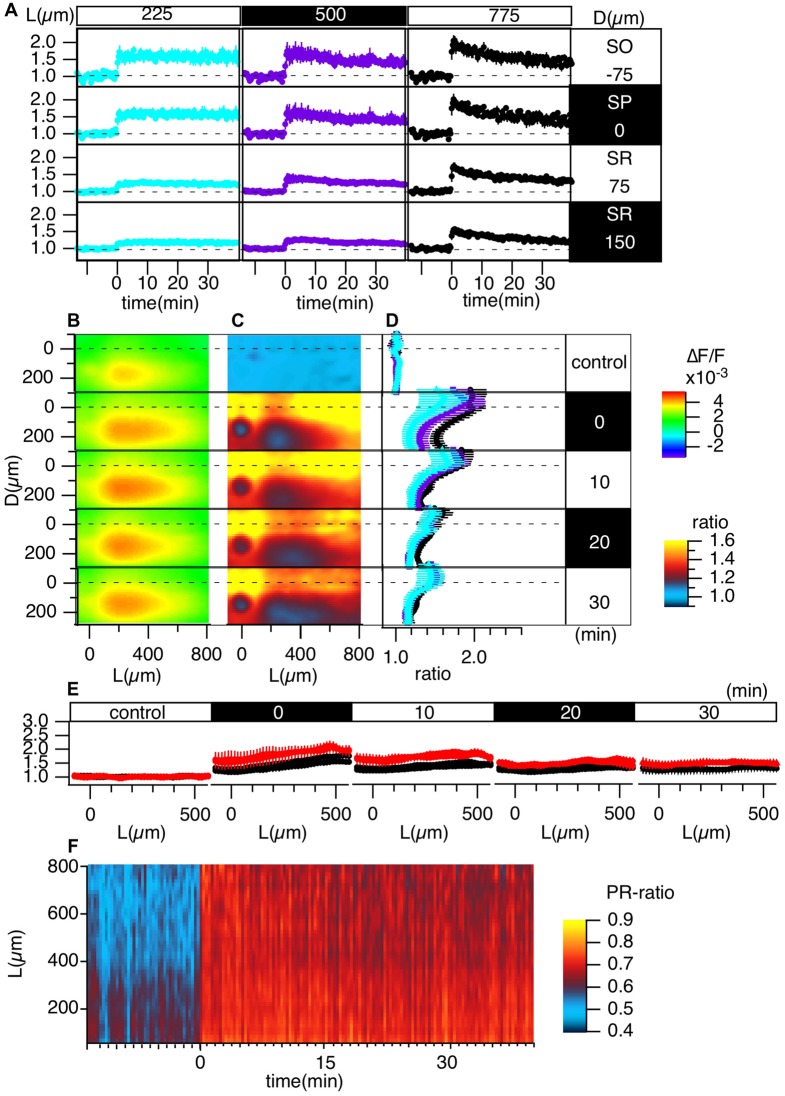
Grouped evaluation of TBS-induced LTP. **(A)** The time changes in the normalized peak amplitude of the optical signals at representative points for three different L positions [*L* = 225 (cyan), 500 (purple), 775 (black) μm] and four different D positions (*D* = −75, 0, 75, 150 μm). **(B)** The peak values mapped in the D-L coordinates. **(C)** The LTP ratio in the D-L coordinates. **(D)** The line profiles of C at different L positions [225 (cyan), 500 (purple), 775 (black) μm] at each time point. **(E)** The line profile along the L axis at different time points corresponding to the control recording and recordings made at 0, 10, 20 and 30 min from TBS. **(F)** The time change in the ratio of the line profiles at the SO (*D* = −75) vs. the SR (*D* = 150), i.e., the PRR, along the L axis. *N* = 4, *n* = 12, bars in **(A,D,E)** indicate SEM.

**Figure 5:**
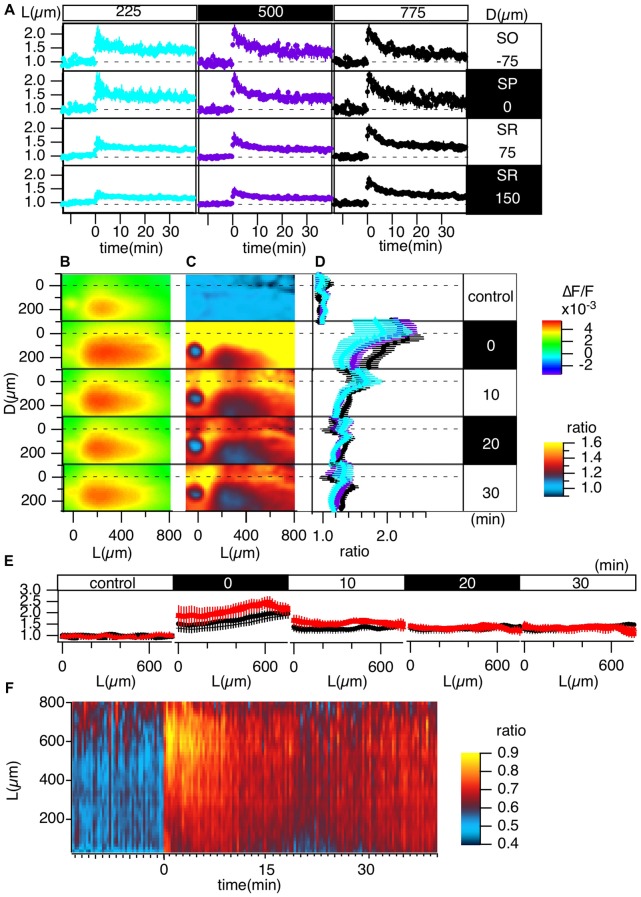
Grouped evaluation of high-frequency stimulation (HFS)-induced LTP. The same analysis of LTP is shown in Figure 3, but the induction protocol is 100 pulses of 100 Hz (HFS). **(A)** The time changes in normalized peak amplitude of the optical signals at representative points at three different L positions [*L* = 225 (cyan), 500 (purple), 775 (black) μm] and four different D positions (*D* = −75, 0, 75, 150 μm). **(B)** The peak values mapped in the D-L coordinates. **(C)** The LTP ratio in the D-L coordinates. **(D)** The line profiles of C at different L positions [225 (cyan), 500 (purple), 775 (black) μm] at each time point. **(E)** The line profile along the L axis at different time sections for control recordings and recordings made at 0, 10, 20 and 30 min from TBS. **(F)** The time change in the ratio of the line profiles at the SO (*D* = −75) vs. the SR (*D* = 150), i.e., the PRR along the L axis. *N* = 2, *n* = 7, bars in **(A,D,E)** indicate SEM.

**Figure 6:**
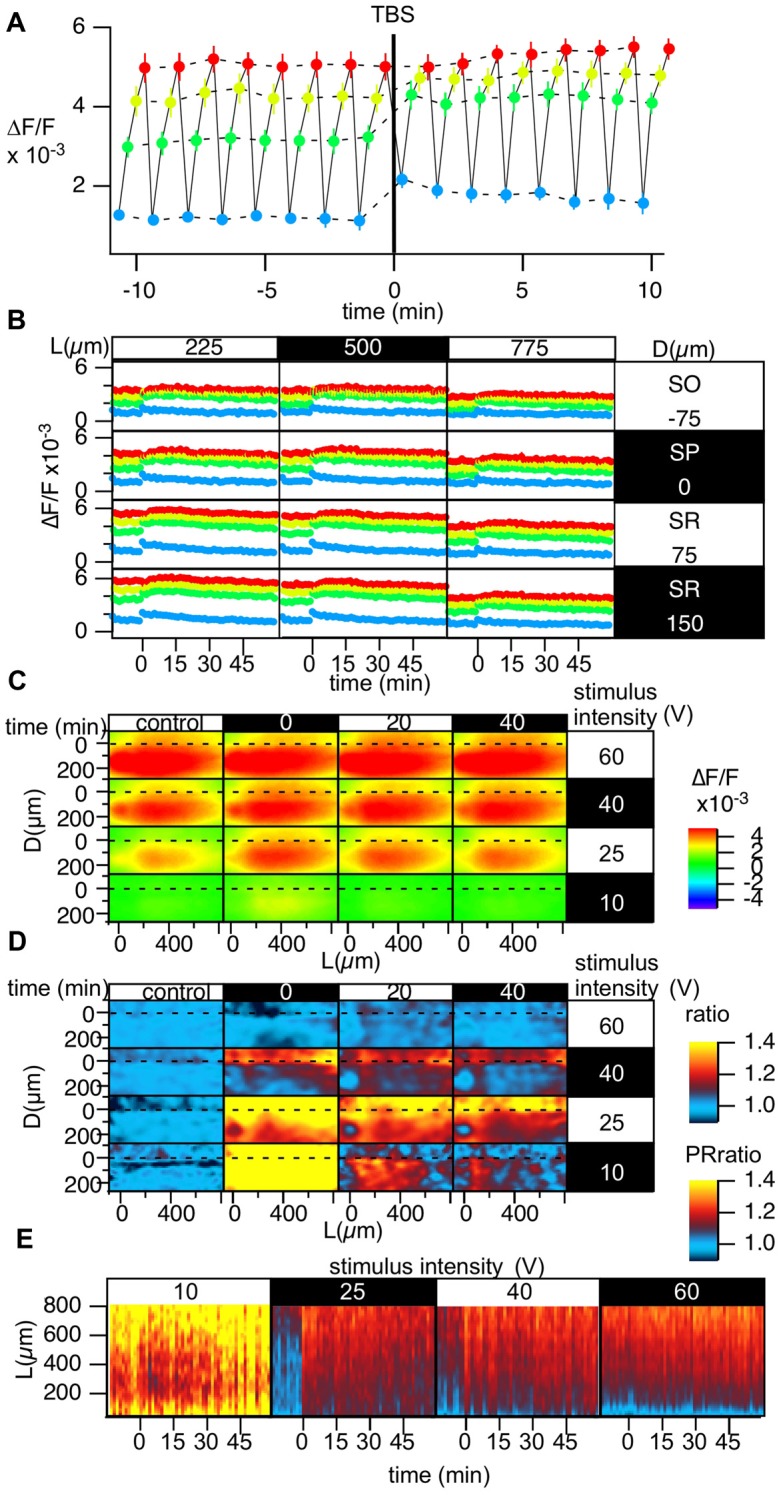
Grouped data of the change in the stimulus-response relationship caused by TBS. **(A)** Change in the optically measured stimulus-response relationship measured by altering the stimulus intensity in four steps c[10 (blue), 25 (green), 40 (yellow) and 60 V (red)] every 20 s while performing optical recording in a time-window containing the induction of LTP in a representative point. **(B)** The time courses of the optical response at the representative points in the D-L coordinates while changing the stimulus intensity; the colors of the points correspond to the stimulus intensity [10 (blue), 25 (green), 40 (yellow), and 60 V (red)]. **(C)** The peak of the optical signals mapped in D-L coordinate at different stimulus intensity and different time. **(D)** The degree of LTP in the D-L coordinate plane corresponding to the stimulus intensity. **(E)** Time change in the PRR for each stimulus intensity. *N* = 2, *n* = 10, bars in **(A,B)** indicate SEM.

A slice sustained with the plexiglass ring was placed in an immersion-type recording chamber. Slices were continuously perfused with ACSF at a rate of 1 ml min^−1^. The ACSF was continuously bubbled with an O_2_/CO_2_ gas mixture. Before placing in the recording chamber, the ACSF was warmed to 31°C with an electronic temperature-controlling heating block (Figure 1). Epifluorescence optics (Tominaga et al., 2000), consisting of two principal lenses [an *f* = 20 mm objective lens (NA = 0.35; Brainvision Inc., Tokyo, Japan) or ×5 MyCAM lens (NA = 0.60; custom made by Olympus) and a ×1.0 Leica Microsystems projection lens], a dichroic mirror (575 nm), absorption (530 nm) and excitation (590 nm) filters, were mounted above the slice. Fluorescence was measured and projected onto a CMOS camera (MiCAM Ultima or MiCAM02, Brainvision Inc., Tokyo, Japan). The ratio of the fractional change in VSD fluorescence to the initial amount of fluorescence (ΔF/F) was used as the optical signal. The frame rate was 0.1 ms/frame on the MiCAM Ultima camera (14 bit ADC, 1.5 × 10^6^ well depth, 80 dB; Figures 2, 3) and 0.6 ms/frame on the MiCAM02 camera (12 bit ADC, 4.5 × 10^5^ well depth, 70 dB; Figures 4–6). The optical signals presented in the following sections are signals that have been spatially and temporally filtered with a Gaussian kernel of 5 × 5 × 3 (horizontal × vertical × temporal directions) two times. The analysis of the optical signals was performed with a procedure developed on the Igor Pro software (WaveMetrics Inc., OR, USA).

A glass microcapillary tube (5-μm opening diameter, filled with ACSF, 1 MOhm) was used as a monopolar stimulating electrode and a recording electrode for field potential recordings. The electrophysiological recording system was controlled with a procedure developed for Igor Pro (WaveMetrics Inc., OR, USA). A 0.05 Hz stimulus was applied to the Schaffer collateral pathway in most of the experimental procedures to monitor synaptic transmission. The IgroPro procedure controlled stimulation patterns through ITC-18 (Instrutech, Longmont, CO, USA) and ESTM-8 (Brainvision, Inc., Tokyo, Japan), and both instruments equip D/A and A/D converters. ITC-18 was used in combination with a linear isolator (A395, WPI Inc., Sarasota, FL, USA; 0–250 μA, 200 μs bipolar), the ESTM-8 has built-in linear isolators to programmatically produce stimulation pulses at different intensities (0–60 V, 300 μs biploar). Neither of these experimental processes interfered with the other. For details regarding the optical recording technique, see our previous article (Tominaga et al., 2000, 2013; Tominaga and Tominaga, 2016).

#### Compensation the VSD Signal

The fluorescence signal from the Di-4-ANEPPS dye was adjusted if needed. Generally, the fluorescence of the dye changes for many reasons (Habib-E-Rasul Mullah et al., 2013). Firstly, one might sometimes experience increased fluorescence during the first 10 min of recordings (Habib-E-Rasul Mullah et al., 2013). We have figured out that this relates to the washout of unbound dye. The dim fluorescence at the first phase of perfusion could be the quenching effect of the dye chemical in the aqueous phase. Washing out the dye prevents this change. The second cause is bleaching. The high intensity of the excitation light and the long duration of the illumination can cause this problem. Hence, the high numerical aperture for collecting light is critical. Our recordings in the present experiments did not produce a measurable bleaching effect, judging by the lack of significant time-change of initial fluorescence intensity. The third one should be caused by unknown factors probably relating to the internalization of the dye. We did not see a significant decrease in fluorescence over 12 h but observed a gradual reduction in the sensitivity of the VSD judged by the intensity of the stimulus artifact at the site of stimulation. The optical artifact showed the gradual decrease by two-time constants, while there was no reason for the reduction of the signal. We compensated the reduction in the data shown in Figures 2, 3 by applying a double exponent function to the artifact intensity. The same analysis on the following measurements did not show any considerable reduction in sensitivity for 2–3 h. This can be dependent on the intensity of the excitation light because the data in Figures 2, 3 recorded at 0.1 ms/frame requires much higher excitation light than the following measurements.

## Results

### The Recording System and the LTP Measured

Figure 1 illustrates the recording chamber system (Tominaga et al., 2000, 2002, 2013) that allows stable, long-lasting fluorescent recording from slices prepared from rodents (rats and mice). We designed a slice chamber that can fix the slice in place while perfusing enough fluid needed to retain the physiology of the slice preparation. The system consists of a thin plastic ring with a polytetrafluoroethylene (PTFE) membrane filter beneath it; the slice sticks to the membrane without any other devices. The device is essential to keep the slice physiology intact for hours and to avoid any mechanical disturbances from recordings. ACSF is perfused from beneath the slice; the fluid then covers the slice towards the suction pipette. The membrane filter helps distribute the solution from the bottom. The tight sealed solution flow channel ensures the supply of the oxygenated solution (Hájos et al., 2009). The membrane filter becomes transparent when wet so that electrophysiological manipulation can be easily performed. The system is also beneficial in reducing the staining solution needed when loading the VSD because the ring can hold a small aliquot (100–110 μl) of staining solution in the incubation chamber. The slices stick to the membrane rather firmly so that mechanical disturbances are avoided; no weights or holding wires are needed to fix the slice in the submerged recording chamber.

Figure 2A shows the recording setup. The excitation light (LEX-2G, Brainvision, Inc., Tokyo, Japan) is equipped with a feedback stabilizing controller to prevent the drift problem, which often occurs in LED illumination (Nishimura et al., 2006). The light is illuminated onto a dichroic mirror and then projected to a water immersion objective lens (×5, NA 0.60, custom made by Olympus and *f* = 20 mm, NA 0.35, Brainvision Inc., Tokyo, Japan). A low-magnification lens with a water immersion objective is suitable to avoid noise from fluid movements while measuring the whole circuit activity. A high NA at low magnification with a substantial focal length (f) requires a large pupil. The combination of two objective lenses with a wide dichroic mirror allows epifluorescence optics with a high numerical aperture. The optics ensured that the excitation light was minimized while capturing a sufficient amount of fluorescence for high-speed imaging (up to 10,000 frames per second, Figure 2B right).

The slice chamber system and the optics established long-lasting, stable recording that could follow the late LTP (L-LTP; Figures 2B–D) induced by a so-called theta burst stimulus (TBS; 10 bursts of 100 Hz in 4 pulses spaced at 200 ms intervals). We routinely measured the stimulus-response relationships in each slice by changing the stimulus intensity programmatically. However, since the stimulus-response relationships differ depending on the distance from the stimulation electrode, we continued to use 100 μA (200 μsec bipolar) stimulation as the control and TBS that gave an almost half-maximum response in the field electrode. Figure 2B shows the representative traces of field excitatory postsynaptic potentials (fEPSPs) and the optical signal at the pixel of the tip of the field electrode (red dot in Figure 2A righthand figure) corresponding to the control recording (black) and recordings made 2 (green), 4 (cyan) and 6 h (blue) from TBS. Figure 2D shows the series of optical signals (data captured at 10,000 frames/s) showing neural propagation along the CA1 area at each time point (control, 2, 4 and 6 h). There was a distinct increase in activation from the control condition to after TBS, but after 2 h, the amplitude and time change of the activation patterns were consistent. The recordings demonstrate that the optical recording system can follow changes in the physiological activity for an extended period of time.

### Normalization of the Optical Signal in the CA1 Area of the Hippocampus by Layers

We transformed the two-dimensional imaging data obtained from the CA1 area to coordinates along the stratum pyramidale (SP). That is, we converted the imaging data in normal x-y coordinates to coordinates corresponding to the distance from the SP layer (D) and the distance from the site of stimulation near the CA1-CA2 boundary (L). We aimed to read the optical signal based on the assumption that each pyramidal cell lies radially on an arc along the following function (Figure 3A).

(1) $${\text{r}}^{2}={\left(\text{x}-{\text{x}}_{0}\right)}^{2}+{\left(\text{y}-{\text{y}}_{0}\right)}^{2}$$

(2) $$\text{x = }({\text{x}}_{\text{s}}-{\text{x}}_{\text{0}})\text{cos}(\text{L/r})-({\text{y}}_{\text{s}}-{\text{y}}_{\text{0}})\text{sin}(\text{L/r})\text{+}{\text{x}}_{\text{0}}$$

(3) $$\text{y}=({\text{y}}_{\text{s}}-{\text{y}}_{0})\text{cos}(\text{L/r})-({\text{x}}_{\text{s}}-{\text{x}}_{\text{0}})\text{sin}(\text{L/r})\text{+}{\text{y}}_{\text{0}}$$

where x_s_, y_s_ indicate the (x, y) coordinate for the site of stimulation.

By fitting the line of the SP in an image to an arc, the parameters of the center (x_0_, y_0_) and radius (r) of the arc were determined. Once these parameters were determined, we converted the CA1 region in the image to a rectangular area shown in the image of Figure 3A. Figure 3B shows an example of a fluorescent image of the CA1 area (Figure 2A) converted to the D-L coordinates. Figure 3C shows the propagation pattern of neuronal activity along the D-L axis. As can be clearly observed in the figure, excitation always ran parallel to the D axis, and excitation began at approximately 150 μm on the D axis.

The conversion (Figure 3B) allows us to evaluate image data from different slices in a normalized manner. Figure 3C represents an example of the propagation pattern of neuronal activity at four different time points (control, 2, 4 and 6 h). The figure shows that the fitting converted the propagation pattern along the Schaffer collateral to the straight line along the L axis. Figure 3D shows the peak values of the optical response at each pixel in the D-L coordinates, and Figure 3E shows the time change of the response for 10 h at pixels at different D-L coordinates. The amount of LTP varies depending on the D-L coordinates. Figure 3F indicates the uneven distribution of the LTP occurrence, where the pseudo-color code indicates the ratio of LTP at each D-L coordinate. Figure 3G shows the line profile of the amplitude of the response shown in Figure 2D at *D* = −75 μm (black; stratum oriens [SO]) and *D* = 150 μm (blue; stratum radiatum [SR]) for the control recording (open symbol) and recordings made at 2, 4 and 6 h from TBS (filled symbol). Figure 3H shows the ratio of LTP, that is, the line profiles in Figure 3F at the same D levels, and time. There was a clear peak in SO at *L* = 775 μm, while the SO signal showed a somewhat steady increase except for in the vicinity of the stimulation. The time course of the response at the representative pixel at *L* = 500 μm SO (*D* = −75 μm) and SR (*D* = 150 μm) and the corresponding point at *L* = 775 μm (Figure 3I) show the changes in the response. The signals in SO reflect the occurrence of the spikes. The results highlight the stability of the dynamics of the neural activity in the circuit- and layer-dependent changes in the occurrence of LTP.

### Grouped Data of TBS-Induced LTP

Figure 4 summarizes the pooled data of the TBS-induced LTP in the D-L coordinates of 12 slices from four animals (*N* = 4, *n* = 12). LTP was more substantial in the SO and SP than in the SR, as shown in Figure 4A. Figures 4C,D demonstrate this phenomenon more clearly. Spatial heterogeneity also appears in the time course of LTP induction depending on the location along the L axis. That is, the LTP could not be clearly observed for smaller L (225 μm) but was more conspicuous for larger L (775 μm). To determine the change in the input-output (I-O) relationship in cells, we calculated the ratio of the peak values at the SO and the SR, which are shown in Figure 4F (i.e., the st. pyramidale-st. radiatum ratio (“PR-ratio”); Tominaga et al., 2009). The PR-ratio changed throughout the entire CA1 area along the L axis when TBS was applied to the Schaffer collateral.

### Comparison of TBS-Induced LTP With HFS-Induced LTP

Several publications highlight the difference between LTP induction and maintenance mechanisms depending on the induction stimulus (Larson and Lynch, 1986; Larson et al., 1986; Huerta and Lisman, 1995; Larson and Munkácsy, 2015). High-frequency stimulus (100 Hz, 1 s) is one of the most common LTP induction stimuli and is known as a “tetanic” stimulus. The maintenance phase of LTP depends on different mechanisms (Korte et al., 1995, 1998; Kang and Schuman, 1996; Smith et al., 2009; Edelmann et al., 2015). We also showed that the induction process of LTP was different depending on the induction stimulus: TBS augmented spike generation, while high-frequency stimulation (HFS) reduced spikes through a GABA-A-dependent mechanism (Tominaga et al., 2000, 2013; Tominaga and Ichikawa, 2002). Here, we compared the differences in LTP in the CA1 area, as shown in Figure 4. Figure 5 illustrates the same experiment as shown in Figure 4 except that the LTP induction stimulus was HFS.

Short-term potentiation is more explicit in HFS-induced LTP over the entire length of the L axis, while short-term potentiation was not clearly observed in TBS-induced LTP (compare Figures 4A, 5A). The heterogeneous distribution of LTP followed a similar trend when comparing TBS-induced LTP with HFS-induced LTP (compare Figures 4B,C to Figures 5B,C). That is, the SO showed a more significant response than the SR. The PRR (Figure 5F) showed a transient increase at the distal (*L* = 775 μm) region, which differs from the TBS-induced change in PRR (Figure 4F).

These results indicate the differences in the spatiotemporal changes in LTP induction depending on the LTP induction stimulus.

### Examination of the Stimulus-Response Relationships During TBS-Induced LTP

The spatial heterogeneity in LTP induction might relate to the I-O relationship of each neuronal element. Hence, we measured LTP induced by four different stimulus intensities (10, 25, 40 and 60 V; Figure 6). Figure 6A represents the optically measured responses at the representative point at the middle of the D-L coordinates. Altering the stimulus intensity every 20 s resulted in the time change of the optically measured responses shown in Figure 6A. TBS (stimulus intensity of 25 V) induced a change in the I-O relationship. Figure 6B shows the same recordings at different points on the D-L coordinates. It is evident that the time course corresponding to a smaller stimulus intensity showed a more significant shift, while a higher stimulus intensity corresponded to a smaller shift (Figures 6C,D). Figure 6D summarizes the degree of LTP at different positions at each stimulus intensity at three different time points. As evident in the figure, for the higher stimulus intensity (60 V), LTP was not significant. At stimulus intensities of 40 and 25 V, the difference between the SR and the SO was evident, and LTP was more substantial in the distal region, as shown in Figures 3, 4. For the smaller stimulus intensity, LTP was more substantial in the vicinity of the stimulus site (Figure 6D). This result may suggest that LTP was conspicuous when the first S-R relation was steep. The heterogeneity of LTP might, at least in part, reflect the original S-R relationship. The change in the PR-ratio was also evident at the middle stimulus intensity but was not evident at the weakest and strongest stimulus intensities (Figure 6E).

## Discussion

We have described and demonstrated an optical recording method with VSD that allows us to record long-term modifications of neural circuits in slice preparations. In the present article, we measured changes accompanied by LTP induction in the CA1 area of mouse hippocampal slices to demonstrate the usefulness of the method. The results indicated that we could follow changes in neural activity in the CA1 area for a long period of time (12 h) with repetitive exposure of the excitation light needed to achieve recording at 0.1 ms/frame for 400 ms. The results show that the method can be successfully applied measure L-LTP (Huang et al., 1996; Kandel, 2001) in slice preparations. Additionally, we showed that the technique can be used to convert images from different slices for grouping according to the morphological background of the CA1 area. The method also highlights the difference in LTP induction by TBS and HFS (Figures 4, 5). It was also shown that the LTP depend on the original stimulus-response relationship (Figure 6). The method will be useful for comparing slices in different test groups, such as genetic modifications, pharmacological treatments and other treatments that can affect hippocampal plasticity changes.

### Optical Recordings From Hippocampal Slices

Since earlier pioneering attempts to apply VSD imaging to mammalian brain tissue (Grinvald et al., 1982), hippocampal slice preparation has been a frequently employed. We can easily assign the optical signals of these preparations to specific membrane elements of the soma and dendrites due to the simple lamellar organization (Witter, 1993). Absorption dyes were often used to show the pharmacological effects of synaptic connections (Ratzlaff and Grinvald, 1991; Barish et al., 1996; Nakagami et al., 1997; Sekino et al., 1997; Kojima et al., 1999; Jin et al., 2002), and a fluorescent dye (Tominaga et al., 2000, 2002, 2013; Mann et al., 2005; Suh et al., 2011; Juliandi et al., 2016; Tominaga and Tominaga, 2016). Absorption dyes (primarily RH482; Momose-Sato et al., 1999; Mochida et al., 2001; Jin et al., 2002; Chang and Jackson, 2006; Chang et al., 2007; Wright and Jackson, 2014) and a fluorescent dye (Tominaga et al., 2000) have also been tried in long-term recordings.

We prefer using Di-4-ANNEPS (Fluhler et al., 1985; Loew et al., 1992) among several other types of fluorescent dyes. This dye is more highly soluble in lipid solutions than in aqueous solutions, which allows the dye to remain longer in the membrane. This characteristic is necessary to achieve long-term recordings, especially for *in vitro* preparations where the physiological solution continuously perfuses the brain slice and causes “washout” of the dye. The washout and unequal staining of slice preparations can cause changes in the signal magnitude that are not due to physiological activity. Fluorescent dyes are advantageous in this regard if the signal is measured relative to the initial amount of light. It is also noticeable that the time-course of the optical signal obtained with Di-4-ANEPPS is different from that recorded with some absorption dye [Grinvald et al., 1982; (RH-155) and Momose-Sato et al., 1999; NK3630 (RH482)]. The absorption dye recordings, especially RH-155, have optical components sensitive to dihydrokainate (DHK; an inhibitor for glial glutamate transporters GLT-1) application and thus include the glial signal (Kojima et al., 1999). The optical signal with Di-4-ANEPPS did not have such components (Tominaga et al., 2002). On the other hand, the absorption dye might have less phototoxicity because of its nature. Hence, one might need to choose which types of dye suit the experiments, depending on the purpose and the possible outcome of the measurements. If the recording system did not cause significant phototoxicity, the fluorescent dye would be better because of less contamination of the glial signal and other intrinsic optical components.

### Implication of Heterogeneity in LTP Induction

There were at least two spatial differences in the degree of LTP. The first is the difference between the SO-SP and the SR, that is, the SO-SP always showed higher LTP than the SR. The SO-SP optical signal mostly reflects the membrane potential events in the soma (Tominaga et al., 2009), which reflect the all-or-none action potential firing property, and thus, that the stimulus-response relationships have a steeper slope than the SR (Tominaga et al., 2000).

The other spatial difference is the L axis, that is, LTP at distal sites is more significant than at proximal sites. The increase in LTP depending on the distance from the stimulating site might reflect the increasing interneuron involvement in the circuit activity. Distant cells receive more inhibition than proximal cells, and this possibility must be tested in future research.

### TBS and HFS

Hippocampal LTP is believed to be a synaptic model of learning and memory (Bliss and Gardner-Medwin, 1973; Bliss and Collingridge, 1993). Tetanic HFS was the first stimulation paradigm that was used to induce LTP in the hippocampus circuit. However, there are arguments that the stimulation pattern is too artificial because such activity is not common *in vivo* in the brain. Rodents show theta activity while exploring novel objects, and the place cell is formed in the phase of these theta oscillation (O’Keefe and Dostrovsky, 1971; Larson and Munkácsy, 2015). The stimulation paradigm that mimics theta activity, i.e., TBS, successfully induced LTP in CA1 synapses (Larson and Lynch, 1986; Larson et al., 1986). TBS is believed to induce different types of LTP (Larson and Munkácsy, 2015).

During the induction phase of LTP, we found that TBS triggers more action potentials but that HFS suppresses action potentials during the stimulus (Tominaga et al., 2002). Both responses involve GABA-a receptor activation (Tominaga and Tominaga, 2010, 2016). The HFS primarily induces long-lasting depolarizing GABA-a potentials (Kaila et al., 1997, 2014; Voipio and Kaila, 2000), where increased conductance inhibits action potential firing by shunting (Tominaga and Tominaga, 2010).

In the present study, LTP induced by TBS and HFS showed different spatiotemporal patterns, and whether or not LTP differs during in the maintenance phase may be important.

### Applicability of the VSD Imaging Analysis of LTP

LTP remains a critical measure for assessing neural function in animal models of neuropsychiatric diseases, including AD and ASD (Monday and Castillo, 2017; Monday et al., 2018). The presented method allows detailed differences possibly caused by pathology and other factors to be shown. The spatiotemporal differences in LTP can introduce arbitrary error, showing differences in the assay that must be avoided.

The spatiotemporal differences in LTP reflect the E/I balance in the circuit. These diseases are closely related to the E/I balance of the circuit (Gogolla et al., 2009; Takesian and Hensch, 2013; Murray et al., 2014). Hence, the spatiotemporal pattern changes during LTP induction (Jackson, 2013; Stepan et al., 2015) could be an important target to measure the pathology of diseases.

Finally, the use of a genetically encoded voltage indicator (GEVI) could significantly advance this field through cell type-specific expression (Vranesic et al., 1994; Knöpfel, 2008, 2012).

## Author Contributions

TT designed the research. YT, MT and TT performed the research. YT and TT developed the software and analyzed the data and wrote the article.

## Conflict of Interest Statement

The authors declare that the research was conducted in the absence of any commercial or financial relationships that could be construed as a potential conflict of interest.
